# Assessment of liver function before major hepatectomy

**DOI:** 10.1093/bjs/znad216

**Published:** 2023-08-02

**Authors:** Pieter Arntz, Pim B Olthof

**Affiliations:** Department of Surgery, Amsterdam UMC, Amsterdam, The Netherlands; Department of Surgery, Amsterdam UMC, Amsterdam, The Netherlands; Department of Surgery, Erasmus MC, Rotterdam, The Netherlands; Department of Surgery, University Medical Center Groningen, Groningen, The Netherlands

## Introduction

The ability of the liver to regenerate after injury or partial loss has enabled surgeons to perform resection of multiple segments in patients with primary or metastatic tumours. Surgical resection of up to 80 per cent of total liver volume is feasible in patients with healthy parenchyma, with acceptable morbidity and mortality^1^. Many patients with primary of secondary liver tumours, however, have hepatic parenchymal dysfunction due to chemotherapy-associated liver injury, cirrhosis, or cholestasis. In these patients, a larger liver remnant is needed to avoid post-hepatectomy liver failure (PHLF). There is no effective treatment for PHLF and it is fatal in 50–90 per cent of patients^2,3^. Strategies to increase the future liver remnant (FLR) include downsizing of the tumour or increasing FLR volume and function^4^. Preoperative portal vein embolization (PVE) of the diseased liver side is the most common strategy to induce hypertrophy of the FLR. Associating liver partition and portal vein ligation for staged hepatectomy (ALPPS) combines (partial) transection of the liver parenchyma with unilateral closure of the portal vein and subsequent completion hepatectomy in the second stage^4^. Preoperative risk assessment is essential before major liver surgery to avoid adverse outcomes. Estimating the volume of the FLR is the most common way to assess the risk of PHLF.

## Liver volume

The calculation of FLR volume can accurately be performed using three-dimensional reconstruction of CT images. Numerous methods using manual, semi-automated, or even automated segmentation can provide the required volume calculations. Many studies have shown that the risk of PHLF is inversely correlated with FLR volume^5^. Despite the wide use of liver-volume measurements and available strategies to increase the FLR, PHLF still remains a significant problem after complex liver surgery. Several studies have addressed the correlation of liver volume with liver function^6^. Especially in patients with compromised liver parenchyma, liver volume does not necessarily correlate proportionally with liver function^7^. Novel techniques to estimate liver function have emerged over the last decade.

## Assessment of liver function

Indocyanine green (ICG) is a fluorescent dye that is exclusively cleared by the liver. After injection, the calculation of its plasma disappearance rate can be used to assess total liver function. Especially in Asia, this is a commonly used test in conjunction with liver volume to estimate the risk of liver failure. Another test for liver function is the methacetin breath test (LiMAx^®^, Humedics GmbH, Berlin, Germany), which also assesses total liver function. This test measures the metabolic turnover of ^13^C-labelled methacetin after intravenous injection. It is less commonly used compared with ICG, but can also be applied to estimate the risk of liver failure when combined with liver-volume assessment^8^. These tests assume a homogeneous distribution of liver function throughout the liver and do not directly quantify the actual FLR function. This may lead to overestimation or underestimation of true FLR function.

Hepatobiliary scintigraphy (HBS) with ^99m^Tc-labelled mebrofenin (^99m^Tc-mebrofenin) or galactosyl human serum albumin (GSA) are methods to quantify total liver function, but also allow the measurement of regional liver function. Using these techniques, any limitations due to inhomogeneous liver function distribution are eliminated. GSA is not registered in Europe and therefore ^99m^Tc-mebrofenin scintigraphy is the scintigraphy method of choice in Western countries^8^.

Mebrofenin is selectively taken up by the liver and excreted in the bile. Labelling with ^99m^Tc allows quantification of the uptake speed by using a γ probe. With single-photon emission computed tomography the uptake in specific regions of the liver can be quantified. By combining the scan with low-dose CT that allows segmentation, the uptake speed of ^99m^Tc-mebrofenin in the FLR can be measured. This is used as a surrogate of FLR function. This regional quantitative measure is usually corrected for body surface area (and expressed as per cent per min per m^2^)^7,8^.

The initial prospective clinical trial in 55 patients who underwent major liver resection identified a remnant liver function of 2.7 per cent per min per m^2^ or higher as a safe cut-off to proceed with liver resection^7^. Follow-up reports from the same centre reported a reduction of PHLF when using this cut-off^9^. External validation studies used similar values as cut-offs for safe liver resection^10,11^.

Although the initial aim was to establish a universal liver function cut-off for safe liver resection, more recent studies have shown that 2.7 per cent per min per m^2^ is insufficient for patients with biliary tumours. These patients who often require major liver resection with biliary reconstruction have high rates of liver failure and mortality. A study of 116 patients undergoing major liver resection with biliary reconstruction for perihilar cholangiocarcinoma showed that a liver function of at least 4.5 per cent per min is a safer strategy in these high-risk patients^12^.

Other high-risk procedures in liver surgery are major resections after PVE or ALPPS. For these procedures, the sequential assessment of liver function has been shown to correlate with outcomes. The increase in liver volume after these procedures is not always proportional to liver function. Therefore, planning of these resections after liver-function measurement has the potential to increase safety^13,14^.

Caution is advised when patients have elevated plasma bilirubin levels. The hepatic uptake transporters of mebrofenin and bilirubin overlap. In the presence of high bilirubin levels the uptake of mebrofenin is usually low, resulting in low liver-function measurements. Whether these truly reflect low liver function or false low function due to competitive uptake cannot be ascertained. Therefore, it is advised not to perform HBS when bilirubin levels exceed 50 µmol/l (2.9 mg/dl) and to be cautious when total liver-function measurements are low^15^. It is also important to mention that there is variation. Liver function may vary to some extent from a scan under similar conditions a week later. Therefore, the binary liver function cut-off should be interpreted in relation to these small variations, as well as to other risk factors for liver failure^16^.

Although dynamic testing has limitations as it only involves surrogate markers of liver function, meaning that they do not provide a direct assessment of all aspects of liver function and are subject to variability and individual differences, estimating liver function with hepatobiliary scintigraphy before major liver resection has been shown to reduce the incidence of PHLF. The technique is simple and can be performed at every nuclear medicine department with the help of practical guidelines currently available^15^.
